# Ezetimibe alone for over 75 years old as a primary prevention to decrease cardiovascular events

**DOI:** 10.1080/07853890.2026.2634484

**Published:** 2026-02-24

**Authors:** Alpo Vuorio, Petri T. Kovanen, Timo Strandberg

**Affiliations:** a Mehiläinen, Aviapolis Health Center, Vantaa, Finland; b Department of Forensic Medicine, University of Helsinki, Helsinki, Finland; c Wihuri Research Institute, Helsinki, Finland; d University of Helsinki and Helsinki University Hospital, Helsinki, Finland; e Center for Life Course Health Research, University of Oulu, Oulu, Finland

**Keywords:** Absorption, ASCVD, cholesterol, ezetimibe, *NPC1L1* gene, old

## Abstract

The EWTOPIA 75 (Ezetimibe Lipid-Lowering Trial on Prevention of Atherosclerotic Cardiovascular Disease in 75 or Older) study supports the reduction in the risk of atherosclerotic cardiovascular disease (ASCVD) events with ezetimibe without statin therapy in persons aged ≥75 years without a history of coronary artery disease. This evidence has also been incorporated into the 2025 Focused Update of the 2019 ESC/EAS Guidelines for the management of dyslipidaemias. What might then be the potential explanations for the observed benefit of ezetimibe to prevent ASCVD in the EWTOPIA 75 study? First, cholesterol absorption efficiency generally increases with age. Therefore, cholesterol absorption-lowering medications, such as ezetimibe, are particularly beneficial in individuals aged 75 years and older, in whom low cholesterol absorption has been associated with fewer recurrent ASCVD events. It is also worth noting that ezetimibe may be more effective than a particular statin in reducing non-high-density lipoprotein cholesterol (non-HDL-C), which includes lipoprotein remnant particles. Therefore, the benefits of the achieved low-density lipoprotein cholesterol (LDL-C) levels are not directly comparable between ezetimibe and statin therapy. It remains to be verified in future studies whether ezetimibe monotherapy would be helpful, specifically in older patients. If so, one possible explanation is that, even in the absence of clinical ASCVD, ezetimibe slows and stabilizes the atherosclerotic process that is still advancing. Finally, a trade-off between a less effective LDL-C lowering but a low frequency of adverse effects and drug interactions could lead to better long-term adherence in the setting of primary prevention.

## KEY MESSAGES

Cholesterol absorption efficiency generally increases with age.

Ezetimibe alone reduces the risk of cardiovascular events in persons aged ≥75 years without a history of coronary artery disease.

## Graphical Abstract

**Figure:**
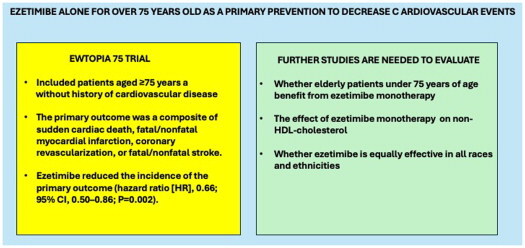

## Introduction

The recent EWTOPIA 75 (Ezetimibe Lipid-Lowering Trial on Prevention of Atherosclerotic Cardiovascular Disease in 75 or Older) open-label and blinded endpoint trial supports the reduction in the risk of atherosclerotic cardiovascular disease (ASCVD) events with ezetimibe without statin therapy in persons aged 75 years or older and without a history of coronary artery disease [1]. This evidence has also been incorporated into the 2025 Focused Update of the 2019 ESC/EAS Guidelines for the management of dyslipidaemias [2]. This guideline mentions that’ new evidence supporting the reduction in the risk of ASCVD events with ezetimibe in the absence of statin therapy (although not necessarily due to statin intolerance) in older persons aged ≥75 years without history of coronary artery disease was provided in the randomized, open-label EWTOPIA 75’. It is noteworthy that the above trial result supports ezetimibe therapy also for those aged ≥75 years without statin intolerance. The EWTOPIA 75 was a multicentre, prospective, randomized, open-label, blinded end-point trial conducted at 363 Japanese medical institutions and in which 3796 participants, male and female, were randomly assigned to use ezetimibe (10 mg once daily) or usual care (Table 1). All participants received dietary counselling and were followed up for approximately 4.1 years. The primary outcome was a composite of cardiac death, myocardial infarction, coronary revascularization, or stroke. This primary outcome was significantly and strongly reduced in the ezetimibe group (hazard ratio [HR], 0.66; 95% CI, 0.50–0.86; *p* = 0.002). There was no difference in the incidence of stroke, all-cause mortality or adverse events between the two trial groups.

**Table 1.** Main points of the study ezetimibe lipid-lowering trial on prevention of atherosclerotic cardiovascular disease in 75 or older [1].

Feature	Study detail
**Study population**	Male and female patients aged ≥75 years and with elevated LDL-cholesterol but without history of CAD were studied at 363 medical institutions in Japan
**Study intervention**	3796 patients (1898 each) were randomly assigned to ezetimibe and dietary counselling or dietary counselling only for three years
**Main outcome**	The primary outcome was a composite of sudden cardiac death, fatal/nonfatal myocardial infarction, coronary revascularization, or fatal/nonfatal stroke
**Main result**	Ezetimibe significantly reduced the incidence of the primary outcome (hazard ratio [HR], 0.66; 95% CI, 0.50–0.86; *p* = 0.002)
**Key strengths**	Provides strong evidence of the ability of ezetimibe monotherapy to prevent cardiovascular events in hypercholesterolemic individuals aged ≥75 years
**Key limitations**	The study was a prospective RCT with blinded outcome assessment, but not a blinded placebo-controlled trial. A number of patients were excluded or lost after randomization The impact of competing risks increased with aging. The study included only Japanese patients

It can be argued that, prior to the EWTOPIA 75 trial, attention had primarily focused on the fact that patients receiving ezetimibe treatment often fail to achieve the LDL-C levels recommended in the guidelines [3]. In addition, the primary target group of ezetimibe has not previously been older adults [4]. Moreover, in such studies the design did not necessarily rule out clinical ASCVD. It has also been highlighted that the combination of statin and ezetimibe is safe, effective, and even preferable for these patients [5–8]. In summary, so far, less attention has been paid to old individuals without ASCVD – i.e. to the primary prevention in this age group. Accordingly, statins have been the primary recommendation for preventing cardiovascular disease among the 70–75+ people [9], and this has mainly applied to secondary prevention. In contrast, RCT evidence in primary prevention has been less clear (CTTC). How cardiovascular prevention for apparently healthy older adults should be carried out, and whether it will help, if implemented, is a matter of current debate [10,11].

## Discussion

How could ezetimibe be effective in ≥75 years old in the setting of primary prevention? Although age is a primary determinant of cardiovascular disease, it is also important to note that older people are biologically heterogeneous [12]. Indeed, cardiovascular conditions are fundamentally related to comorbidities and frailty, two things that vary greatly from one individual to another. So, the persons without ASCVD events at the age of ≥75 years most probably have better cardiovascular condition compared to many of their counterparts.

However, because of their age, there is nevertheless a significantly elevated absolute risk of future cardiovascular events. In fact, 75 years is commonly considered the beginning of old age, with relevant—but not necessarily clinically manifest—bodily changes due to ageing [12,13]. It has been challenging to tailor ASCVD prevention in these special, individualized circumstances, in which the likelihood of a heightened risk-benefit ratio is real. As part of the ageing process, the propensity to endothelial dysfunction increases, further exacerbated by age-dependent alterations in the fibrinolytic cascade and systemic inflammation [14]. Part of the challenge is also the potential problems caused by overlooked age-related alterations in the absorption and metabolism of drugs and in drug interactions, which can be at least partly because patients aged 75 years or older, have only recently received due attention in the dyslipidaemia guidelines.

There has been a scarcity of RCTs among older patients. Still, the situation is improving for primary prevention, and 2 large RCTs comparing atorvastatin 40 mg with placebo, specifically in older individuals, are ongoing [15–17]. Among the earlier studies, the Pravastatin in Elderly Individuals at Risk of Vascular Disease (PROSPER) RCT included patients aged 70–82 years and demonstrated the benefit of pravastatin therapy in reducing ASCVD events [18]. Since then, several cohort studies of statin therapy have been conducted among patients aged 75 years or older [19–21]. A study by Kim et al. [22] suggested the benefit of statin primary prevention among 75+ individuals, which is in line with a meta-analysis [23]; however, this is opposite to that of Ramos et al. [19]. Interestingly, in the study by Kim et al. [22], the mean LDL-C level achieved with statin therapy was 2.17 mmol/L (84 mg/dL), i.e. relatively high. But in the EWTOPIA 75, the mean LDL-C level achieved was even higher: from 4.2 mmol/L (162 mg/dL) at baseline to 3.1 mmol/L (120 mg/dL) in the ezetimibe group [1].

What can then explain the benefit of ezetimibe to prevent ASCVD in the EWTOPIA 75 study? Although there is no single, clear explanation, several potential benefits of ezetimibe treatment can be identified. While in the EWTOPIA 75 study, the LDL-C level was clearly higher than in the statin primary prevention study by Kim et al. [22], it is worth noting that when combined with a statin, ezetimibe is potentially more effective in decreasing non-high-density lipoprotein level (non-HDL-C), which includes also the lipoprotein remnant particles, as recently demonstrated by Cha et al. [6]. Therefore, although similar LDL-C levels were achieved, the combination of ezetimibe, as a lipoprotein remnant-lowering drug, and a moderate-intensity statin, also capable of lowering the level of lipoprotein remnants, could jointly decrease the level of total and non-HDL cholesterol levels more than the high-intensity statin therapy alone [6,7,24]. When added to statin therapy, ezetimibe effectively reduces LDL-C levels in patients regardless of ethnicity [25]. However, the Japanese population may have some unique ethnic characteristics that could have contributed to the ezetimibe response. This possibility is supported by data showing differences in the frequency of the *NPC1L1* polymorphism between Japanese and, for example, Canadians [26]. As a note, the baseline LDL-C level was relatively high in the EWTOPIA 75 Trial (approximately 4.2 mmol/L), which may have attenuated the benefit in those with lower LDL-C levels [27]. Unfortunately, no direct comparisons between the effects of ezetimibe and statin monotherapy on non-HDL-C levels appear to be available. Of note, there are no clinically significant effects of age on ezetimibe pharmacokinetics, and no dosage adjustment is necessary in cases of mild hepatic impairment or mild-to-severe renal insufficiency [28]. Polypharmacy is a widespread issue among older adults, with its prevalence rates ranging from 25% to 52% in Europe [29]. Overall, ezetimibe has a favourable drug interaction profile, and, fortunately, it lacks relevant interactions with other drugs commonly used in patients with hypercholesterolaemia [28].

What about the effect of intestinal cholesterol absorption on atherosclerosis? It has been shown that not only the level of plasma LDL-C but also the balance between cholesterol absorption and synthesis is associated with atherosclerosis [30–33]. Importantly, in patients not on lipid-lowering agents, low serum lathosterol level, as a marker of low whole-body cholesterol synthesis, was associated with an excess of fatal cardiovascular events and of mortality [34]. However, regarding the magnitude of cholesterol absorption, there are conflicting results regarding its contribution to atherosclerosis, which may be partly because most of the studies have been carried out in patients with, for example, either hypercholesterolemia or diabetes, i.e. two conditions in which the metabolic disease itself may have confounding effects on the results [35]. In the near future, it could be possible to define individual genetic metabolomic fingerprints to analyse better cholesterol metabolism, *i.e.* including both cholesterol synthesis and absorption [36]. But this task is challenging because cholesterol absorption alone involves several genes and their complex network [37]. In the Singapore Chinese Health Study (*n* = 21,873), analysing dietary cholesterol consumption and genetically quantified cholesterol absorption, ASCVD mortality was associated with a greater genetically defined magnitude of cholesterol absorption [38]. Importantly, Helgadottir and coworkers [39] conducted a large genetic study to determine whether variability in dietary cholesterol and phytosterol absorption would modify the risk of coronary artery disease. They concluded that high-cholesterol absorbers may benefit from the combined effects of ezetimibe treatment and a reduction in dietary cholesterol intake. Thus, it would be ideal to individualize ezetimibe therapy based on each patient’s cholesterol absorption status [40]. Moreover, ezetimibe’s good tolerability, i.e. fewer side effects than with statins, enhances both personalized efficacy and treatment adherence [41]. The combination of ezetimibe with a moderate-intensity statin is effective in reducing LDL-C levels [42]. Importantly, in a study population aged around 66 years, a combination consisting of ezetimibe and a moderate-intensity statin, previously shown to be effective in a population with high cholesterol absorption, was as effective as high-intensity statin monotherapy in preventing cardiovascular events [43]. Unfortunately, no separate analysis of the cost-effectiveness of ezetimibe monotherapy (i.e. without a statin) is available.

But the association between cholesterol absorption and ASCVD may not be straightforward. In an observational study, low cholesterol absorption (reflected by lower serum cholestanol/cholesterol ratio) was associated with fewer recurrent ASCVD events among cardiovascular patients aged 75 years and older, thus giving support to ezetimibe effects [44]. This in contrasted by the results in the Kyushu Elderly Ezetimibe Phytosterol (KEEP) study among individuals aged 75 years or older [45]. In that study, higher baseline serum campesterol levels (an indicator of cholesterol absorption) were associated with a lower risk of ASCVD events. However, the therapeutic effect of ezetimibe did not depend on serum campesterol levels, and, accordingly, this observation did not support the idea that the benefit of ezetimibe is based on different baseline cholesterol absorption in older patients. The authors concluded that further research is needed to determine whether measurements of cholesterol absorption/synthesis can be used in relation to ezetimibe treatment. There are probably mechanisms beyond lowering LDL-C levels with ezetimibe that are associated with the observed decrease in ASCVD incidence [45]. Thus, the KEEP study found that the cardiac marker N-terminal pro-B-type natriuretic peptide, but not the inflammatory marker hs-CRP, was significantly associated with the risk of cardiovascular events [45]. Cholesterol metabolism decreases with aging, but cholesterol synthesis, as determined by the lathosterol/cholesterol-ratio, does decrease with aging and cholesterol absorption markers did not clearly decrease with aging [45]. Thus, the therapeutic aim for lowering cholesterol differs between middle-aged and elderly adults.

An additional interesting piece of information comes from a recent study focusing on the variation of the Niemann-Pick C1-like 1 (*NPC1L1*) gene [46]. In this study, five genetic variants in *NPC1L1* were analysed among 5804 older participants from the PROSPER trial. It was shown that *NPC1L1* genetic variation was associated with LDL-C levels and ASCVD risk after adjusting for apolipoprotein E phenotype. Additionally, genetic variation in the *NPC1L1* gene may alter the response to ezetimibe treatment. Indeed, it is well established that mutations that disrupt the proper function of NPC1L1, as the significant cholesterol absorption-mediating molecule in the intestinal wall, are associated with reduced plasma LDL cholesterol levels and a lower risk of ASCVD [47].

Taken together, ezetimibe treatment has numerous beneficial cardiovascular effects. It is, however, unclear how each effect is related to age. The numerous beneficial effects of ezetimibe include moderate lowering LDL-C levels, maintaining of endothelial function, and it has a beneficial impact on the fibrinolytic activity, reduces inflammation, promotes the homeostasis between the oxidant and antioxidant activities, and, finally, as a result of the above-listed antiatherogenic effects, it may even decrease the volume of atherosclerotic plaques with ensuing increase of their stability [48–51].

## Conclusions

According to one RCT and some theoretical considerations, ezetimibe single therapy is beneficial in the primary prevention of cardiovascular disease in individuals aged 75 years or older. Also, the fresh European Dyslipidaemia guidelines recommend ezetimibe therapy for this purpose. However, more studies are needed to elucidate the mechanisms of benefit. In individuals aged 75+, one possible explanation for the beneficial effects of ezetimibe on cardiovascular health in the primary prevention setting is that, despite no clinical ASCVD, atherosclerosis is still advancing solely due to advanced age, but in a subclinical, stable way. Finally, a trade-off between less effective LDL-C lowering and a low frequency of adverse effects with ezetimibe single therapy could lead to better long-term adherence in primary prevention [52].

## Data Availability

Data sharing does not apply to this article as no new data were created or analysed in this study.
